# Constellation Map: Downstream visualization and interpretation of gene set enrichment results

**DOI:** 10.12688/f1000research.6644.1

**Published:** 2015-06-24

**Authors:** Yan Tan, Felix Wu, Pablo Tamayo, W. Nicholas Haining, Jill P. Mesirov

**Affiliations:** 1 Broad Institute of MIT and Harvard, Cambridge, MA, 02142, USA; 2 Bioinformatics Program, Boston University, Boston, MA, 02215, USA; 3 Department of Pediatric Oncology, Dana-Farber Cancer Institute and Harvard Medical School, Boston, MA, 02215, USA; 4 Division of Hematology/Oncology, Children’s Hospital, Harvard Medical School, Boston, MA, 02215, USA

**Keywords:** gene set enrichment analysis, GSEA, gene expression, signature, pathway, visualization, mutual information

## Abstract

**Summary:** Gene set enrichment analysis (GSEA) approaches are widely used to identify coordinately regulated genes associated with phenotypes of interest. Here, we present Constellation Map, a tool to visualize and interpret the results when enrichment analyses yield a long list of significantly enriched gene sets. Constellation Map identifies commonalities that explain the enrichment of multiple top-scoring gene sets and maps the relationships between them. Constellation Map can help investigators take full advantage of GSEA and facilitates the biological interpretation of enrichment results.

**Availability:** Constellation Map is freely available as a GenePattern module at http://www.genepattern.org.

## Introduction

Gene set enrichment analysis (GSEA) ( Mootha *et al.*, 2003; Subramanian *et al.*, 2005) is widely used to analyze transcription data by identifying sets of genes that are coordinately up- or down-regulated in a phenotype of interest. By focusing on cumulative changes in the expression of multiple genes, GSEA can detect biologically meaningful processes (e.g., groups of genes in the same pathway) that differ significantly between phenotypes. The broad use of GSEA has, however, resulted in a rapid increase in the number of gene sets available for analysis. This presents a new challenge, because, depending on the collection(s) of sets employed, GSEA may yield tens or hundreds of significantly enriched gene sets. Thus, investigators may face the difficult task of sifting through multiple high scoring gene sets to find biologically relevant relationships between them. To address this need we developed Constellation Map, a network-based visualization tool, to facilitate the downstream analysis of enrichment results.

## Description & case study

Constellation Map presents gene set enrichment results generated by GSEA as a radial plot. Each node of the plot represents a significantly enriched gene set. Nodes that are closer to the origin (i.e., with shorter radial distance) are more highly associated with the phenotype of interest. The angular distance between two nodes represents the per-sample similarity of their respective gene sets’ enrichment. We use a normalized mutual information (NMI) score to measure both these associations (see Workflow & Methods). Edges between nodes denote an overlap between sets’ member genes, while edge thickness captures the relative size of the overlap.

These elements are all presented via a JavaScript-powered browser environment for interactive exploration. Investigators can quickly, visually identify tight clusters of connected nodes, i.e., gene sets with similar enrichment patterns that may represent different aspects of the same biological process, and assess how similar each node is to the others in that cluster. Identified clusters may be further interrogated by selecting them, extracting overlapping genes, and querying those genes using a variety of functional annotation tools (MSigDB, GeneMANIA, and DAVID) ( Dennis *et al.*, 2003; Subramanian *et al.*, 2005; Warde-Farley *et al*., 2010) all within the tool. Constellation Map thus accelerates the biological interpretation of enrichment results by clarifying the relationships of high scoring gene sets relative to the phenotype and relative to each other.

**Figure 1.**
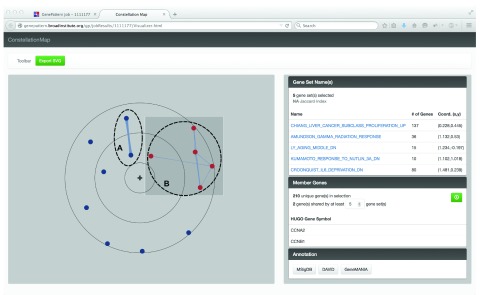
Screenshot of the Constellation Map visualizer. Here we show the JavaScript powered Constellation Map visualization of the top 13 gene sets significantly associated with the transcriptional response in PBMC to vaccination with trivalent inactivated influenza vaccine (TIV). Nodes represent gene sets, and edges indicate overlap of member genes with thickness proportional to the amount of overlap. Gene sets radially closer to the origin are more highly associated with the high response phenotype. Gene sets in close angular proximity have similar enrichment patterns. Visually identified clusters were enriched for (A) immunoglobulin and (B) proliferation genes. Proliferation cluster nodes have been selected (highlighted in red), and the relevant gene set names, overlapping genes, and other metadata are displayed in the side panel.

We previously demonstrated these advantages by applying our tool to uncover gene sets that characterize the transcriptional response to trivalent inactivated influenza vaccine (TIV) ( Tan *et al.*, 2014). We analyzed expression profiles of peripheral blood mononuclear cells (PBMCs) from 24 subjects vaccinated with TIV and performed enrichment analysis to discriminate high and low responders. We used Constellation Map to project 13 gene sets significantly associated with high response (FDR < 0.25) ( Figure 1). We identified two distinct clusters of gene sets enriched for immunoglobulin (labeled A) and proliferation genes (labeled B) and showed that these sets are tightly associated with the immune response to TIV. Visualizing and annotating with Constellation Map was crucial to our identification of the common biological processes that resulted in enrichment of these gene sets.

## Advantages of Constellation Map

Several visualization and interpretation tools have been developed over the last few years to address the challenge of downstream interpretation of enrichment results. Unlike some of these tools, which are designed to use Gene Ontology (GO) or other hierarchically organized gene sets ( Grossmann *et al.*, 2007; Lewin & Grieve, 2006), Constellation Map can also perform well with gene sets derived from larger, less structured collections, such as the pathways and experiment signatures found in the popular MSigDB collections ( http://www.msigdb.org). The network-based visualizer, Enrichment Map (EM) ( Merico *et al.*, 2010), is somewhat similar to Constellation Map in that it displays gene set enrichment results using a network representation where nodes represent sets and edges represent gene overlap between sets. However, EM clusters gene sets based on member gene overlap regardless of their relationship to the phenotype of interest. This ignores the possibility of gene sets having similar enrichment profiles despite little member gene overlap. Conversely, EM could highlight gene sets with some overlap that are different in their enrichment profiles across a group of samples. Constellation Map, on the other hand, takes similar per-sample enrichment profiles into account, providing this information to the investigator as an intuitive angular distance.

## Workflow & methods

**Figure 2.**
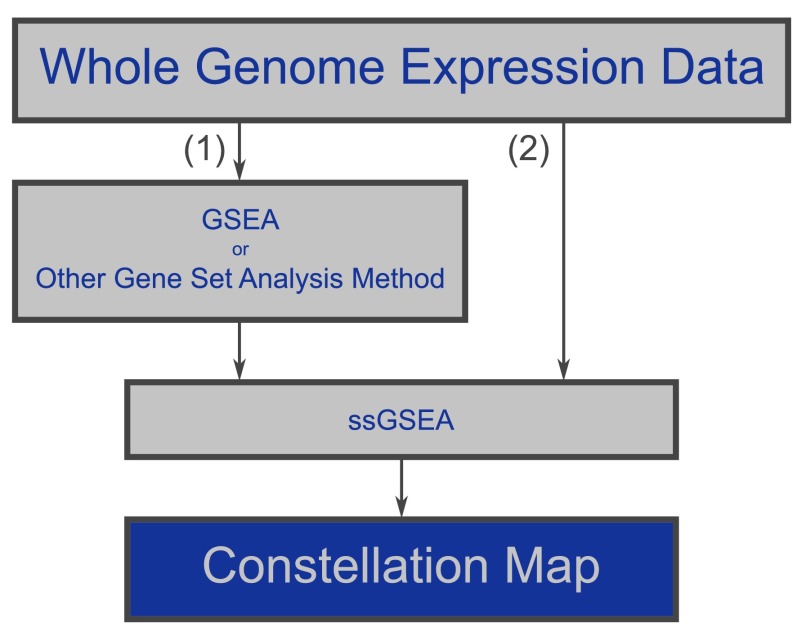
Diagram depicting the Constellation Map workflow. Two options exist. Users may either (1) analyze their whole genome transcript expression data using a preferred analysis method (e.g., GSEA) to identify a group of top-scoring gene sets, project samples into the space of these top-scoring gene sets using ssGSEA, and visualize the results using Constellation Map or (2) directly project their data into the space of all gene sets of interest using ssGSEA and choose only a small group of these gene sets to display with Constellation Map.

A user begins the Constellation Map workflow ( Figure 2) by either: (1) identifying a group of top-scoring gene sets using GSEA, or some other preferred enrichment analysis approach, and utilizing single sample gene set enrichment analysis (ssGSEA) ( Barbie *et al.*, 2009) to project samples into the space of top-scoring gene sets; or (2) directly projecting data into the space of all gene sets of interest using ssGSEA and later choosing to display only those most associated with a phenotype. The gene set enrichment projection result from this module is used as the input for Constellation Map. ssGSEA is an extension of GSEA, available as a GenePattern module *ssGSEAProjection* ( http://www.genepattern.org), that generates an enrichment-ranked list of gene sets for each sample.

Using the gene set enrichment scores obtained via ssGSEA, Constellation Map estimates the probability density functions of gene set and phenotypic class variables using a kernel density estimation. These density functions are subsequently used to calculate NMI scores for each gene set, which capture the association between each gene set’s enrichment scores and phenotypic classes ( Equation 1). The NMI of two variables is their mutual information ( Equation 2) divided by their joint entropy ( Equation 3) ( Shannon, 1948). We chose to use the NMI metric because it is independent of the sample distribution and more sensitive to nonlinear associations than the more commonly used correlation coefficients. As NMI is unidirectional, we created a signed version (SNMI) using the sign of the Pearson correlation to distinguish between positive and negative associations ( Equation 4).

$$\begin{array}{l} {}_{Information\;\left(NMI\right)}^{Normalized\;Mutual}\;NMI(x,y)=\frac{MI(x,y)}{H(x,y)}\;(1)\\ Mutual\;Information\;MI(x,y)=\iint P(x,y)log_{2}\frac{P(x,y)}{P(x)P(y)}dxdy\;(2)\\ \;Entropy\;H(x)=–\int P(x)log_{2}P(x)dx\;(3)\\ {}_{\underset{\left(SNMI\right)}{Mutual\;Information}}^{Signed\;Normalized}\;SNMI(x,y)=sign(\rho (x,y))NMI(x,y)\;(4)\\ Objective\;Function\;\sigma (X)=\sum_{i<j}{\left(\delta_{ij}–d_{ij}\right)}^{2}\;(5) \end{array}$$

After calculating the NMI scores, gene sets that significantly associate with phenotypes of interest can be selected (using an FDR or NMI score cutoff) and projected onto a radial plot. A second set of NMI scores is calculated pairwise across the *N* selected gene sets to estimate the similarity between their ssGSEA enrichment profiles. These pairwise NMI scores are converted into dissimilarity scores, *d = 1* - *NMI*, which provides a true distance metric ( Vinh *et al.*, 2010). Constellation Map uses this property to construct an *N*-by- *N* distance matrix *D* containing the distances *d* between all pairs of gene sets. Constellation Map then projects the distance matrix onto a radial plot using the multidimensional scaling projection R package “SMACOF,” version 1.5-0 ( Leeuw & Mair, 2009). An angular distance matrix Δ is calculated by minimizing the objective function ( Equation 5), where *δ _ij_* is the angular distance and *d _ij_* is the original distance (stored in *D*) between gene sets *i* and *j*. The gene sets are plotted as points distributed about the origin. Angular distance between two gene sets is determined from Δ and is proportional to the similarity of the gene sets’ enrichment profiles. Radial distance (i.e., distance to the origin) indicates the gene set’s association with respect to the phenotype ( *1* - *NMI*).

The final step of Constellation Map projection involves calculating pairwise Jaccard indices across the gene sets. The Jaccard index is equal to the number of genes shared by two sets divided by the number of genes in their union. For pairs with Jaccard indices greater than a given threshold, edges are drawn connecting the respective nodes where the thickness of each edge is proportional to the Jaccard index ( Jaccard, 1901; Merico *et al.*, 2010).

## Summary

Constellation Map is a powerful and intuitive tool in that it allows investigators to determine the relevance and relationships of their gene sets with relative ease. The visualizer evaluates a large set of gene set enrichment profiles using a variety of comparison metrics and presents these metrics in an understandable manner. This uncluttered, simple presentation reduces an investigator’s workload by easing the complex task of having to interpret the enrichment profiles of many gene sets. Just as Constellation Map aided us in identifying subgroups of gene sets with distinct immunologic biologies in our TIV vaccination case study (see above), we believe that investigators can similarly enhance their enrichment analyses by leveraging Constellation Map across their own data, helping them to draw meaningful biology from their many gene sets.

As the scientific community continues discovering new regulatory pathways, perturbation signatures, etc. and casting them into lists of genes, gene set collections will continue to expand. This growth may complicate the historically straightforward enrichment analysis when results contain thousands of gene sets, many of which may be redundant or related. Thus, there is a real need for downstream tools that can elucidate the major biological processes represented in these results and present them in an informative, exploratory manner. Constellation Map, with its mutual information-based layout, interactive visualizer, and connection to annotation services is well suited to meet this need.

## Data availability

TIV vaccine gene expression datasets are available from the NCBI Gene Expression Omnibus; accession number GSE29619 ( Nakaya *et al.*, 2011). Gene sets are contained in MSigDB version 3.0, collection C2 ( Subramanian *et al.*, 2005) available at the MSigDB download page ( http://www.msigdb.org).

## Software availability

Constellation Map is freely available as a GenePattern module ( http://genepattern.broadinstitute.org/gp/pages/index.jsf?lsid=urn:lsid:8080.gpbroad.broadinstitute.org:genepatternmodules:345). Module source code is available at Zenodo (doi: 10.5281/zenodo.18586) and is maintained at the GenePattern community module archive, GParc ( http://www.gparc.org); this module may be installed on a private GenePattern server (R-3.0 and Java required). Proper rendering of the visualizer requires a modern, JavaScript-enabled web browser; the authors recommend using the latest versions of Firefox or Chrome. Constellation Map is distributed under the open source MIT License.

Constellation Map is freely available as a GenePattern module ( http://genepattern.broadinstitute.org/gp/pages/index.jsf?lsid=urn:lsid:8080.gpbroad.broadinstitute.org:genepatternmodules:345). Module source code is available at Zenodo (doi: 10.5281/zenodo.18586) and is maintained at the GenePattern community module archive, GParc ( http://www.gparc.org); this module may be installed on a private GenePattern server (R-3.0 and Java required). Proper rendering of the visualizer requires a modern, JavaScript-enabled web browser; the authors recommend using the latest versions of Firefox or Chrome. Constellation Map is distributed under the open source MIT License.
