# Supplementary material for: Constellation Map: Downstream visualization and interpretation of gene set enrichment results
Source: F1000Res. 2015 Jun 24;4:167. [Version 1] doi: 10.12688/f1000research.6644.1 (PMC4648224; doi:10.12688/f1000research.6644.1)
Supplement: Peer review history

# Peer review: Referee response for version 1

**DOI:** 10.5256/f1000research.7138.r10711

**Published:** 2015-10-07

**Authors:** Sayan Mukherjee

**Affiliations:** 1 Department of Statistical Science, Duke University, Durham, NC, USA

Exploratory data analysis, visualization, and hypothesis generation in genomics are essential tools. Methods and software that are clear, robust, and easily accessible are rare and very important to biomedical researchers. I found the approach described in Constellation Map intuitive and visually appealing. The statistical methods were reasonable, I would have liked to also see the possibility of metrics based on likelihood models, the kernel density estimation does go a bit in that direction.

I was able to use the software. I would have liked for there to be an R version to download from cran as well as some vignettes independent of GenePattern. That said this work is useful and in my opinion some thought went into the visualization.

I have read this submission. I believe that I have an appropriate level of expertise to confirm that it is of an acceptable scientific standard.

# Peer review: Referee response for version 1

**DOI:** 10.5256/f1000research.7138.r9357

**Published:** 2015-07-07

**Authors:** Hilary Coller

**Affiliations:** 1 Department of Molecular, Cell and Developmental Biology, University of California Los Angeles, Los Angeles, CA, USA

The software that Mesirov and colleagues developed is designed to help scientists interpret the results of their gene set enrichment analysis results.  They have developed a useful tool for visualizing the different datasets that emerge and organizing them with relation to each other. 

The software organizes the gene sets so that those that are most relevant are close to the origin and those that have similar patterns are close to each other. Those with overlapping datasets are indicated with lines between the dots.  The metrics used by the software were well-selected, and the visualization approach should make it intuitive for users to gather valuable information about their data.  Use of this software will likely allow scientists to gain more biological insights from GSEA analyses of their datasets.

I have read this submission. I believe that I have an appropriate level of expertise to confirm that it is of an acceptable scientific standard.
